# Post exercise ice water immersion: Is it a form of active recovery?

**DOI:** 10.4103/0974-2700.66570

**Published:** 2010

**Authors:** Fatimah Lateef

**Affiliations:** Senior Consultant, Director of Undergraduate Training and Education, Department of Emergency Medicine, Singapore General Hospital, Singapore

**Keywords:** Ice water immersion, contrast temperature water therapy, delayed onset muscle soreness, athlete

## Abstract

Ice water immersion and contrast temperature water immersion therapy post exercise is fast becoming a common practice among athletes involved in a variety of sports. Several mechanisms have been put forth to explain the rationale for its use. However, there is still a lack of evidence from a sufficiently large-scale trial to support the routine practice and formal incorporation into certain sporting guidelines. We describe here two athletes who applied the therapy post exercise and presented to the Emergency Department with delayed onset muscle pain.

## INTRODUCTION

Water has been used in many forms of therapy and one of these includes ice water immersion therapy post exercise. Taking a post exercise plunge into an ice bath at 12–15°C appears to be a common practice among many elite athletes. This is believed to be a practice that would reduce muscle pain and soreness after training sessions and competitions. Some athletes prefer to use contrast water therapy, i.e. alternating immersion in cold and warm water. Many use either ice bath immersion for a period of 5–10 min (sometimes reported up to 20 min) or alternating therapy between the ice bath plunge and tepid water immersion, each lasting 1–5 min.

Despite this being a practice among some athletes, especially elite ones, it is not an evidence-based recommendation and studies performed thus far have been small or inconclusive. Some have argued against this practice as it offers no benefits for pain, swelling or isometric strength and function. In fact, some observations have been made to show that this practice may cause muscle soreness the day after. Perhaps it is the placebo effect that is being utilized.[1–8]

Currently, the possible mechanisms postulated for the use of cold water immersion therapy post exercise include:[1–4]

1. With intense exercise, there will be some microtrauma and tears in the muscle fibers affected. This muscle damage will stimulate muscle cell activity (hypertrophy in the long term) and help in the repair and strengthening of the muscle. This is also thought to be the explanation for the delayed onset pain and soreness (delayed onset muscle soreness), which often presents 12–72 h post exercise.
2. The ice bath will cause constriction of blood vessels. This has been suggested as a mechanism that helps with the flushing of waste products, such as lactic acid, out of the affected tissue.
3. With the cold temperature, there will be a reduction of the metabolism and this can cause a slowing down of the physiological processes.
4. The cold temperature will reduce swelling and tissue breakdown.
5. Ice water immersion is also said to be able to shift lactic acid.

## CASE REPORT

MS, a right hand-dominant, 23-year-old, male athlete involved in martial arts, was brought to the Emergency Department (ED) for bilateral arm pain. He presented one day after his last match and this was his first such presentation. He had been involved in the sport for the last 5 years. He had taken a dip in a tub filled with ice cubes immediately upon completion of the match. This was a practice he started 6 months ago. Physical examination was normal. There was mild tenderness over the biceps and forearm muscles of the right upper limb. Although he had pain in the left arm, there was no tenderness.

NCK is a 25-year-old male marathoner. He came to the ED for lower limb pain 12 h after completing a marathon. He had pain over his calf muscles as well as quadriceps bilaterally. He could walk but had pain as he tried to squat or get up from a sitting position. He usually had some degree of such discomfort after a long run but this was the worst he had encountered. NCK mentioned that he had dipped himself into a tub of ice immediately after he completed the 42-km run in 2.5 h. Examination showed no external injuries and the muscle power was normal in all groups, as was the neurological examination.

Both men were fit young athletes with no medical history. They were well conditioned and undertook regular training. They both also claimed to have used the ice water immersion therapy post exercise. Both did this regularly and found it to be a practice that helped them recover more quickly from “muscle soreness and tiredness.”

## INVESTIGATIONS

Both young athletes had their full blood count, renal panel, electrolytes and creatine kinase (CK) levels examined. The results of all the investigations were normal. The CK levels were in the normal range in MS and showed a slight elevation in NCK (199 U/L, normal range being 38–160 U/L).

Both their electrocardiograms were normal. Urinalysis was also normal and did not show gross or microscopic hematuria. Radiography of the affected areas was normal.

## MANAGEMENT

Both men were given mild analgesia and muscle relaxant and advised on adequate rest before commencing training again. They were to increase their fluid intake and monitor the color of their urine. They were also informed to discuss their return to exercise and training as well as their ice water immersion practice with their respective coaches.

## DISCUSSION

Recovery from exercise is crucial for athletes, especially with repeated bouts of exercise. In competitive situations, where athletes compete numerous times over several days, enhancing recovery may prove a competitive advantage. Immersion in water is a practice that appears to be catching on among many athletes. Water immersion may cause physiological changes in the body, such as intracellular–intravascular fluid shift, reduction of muscle edema as well as increased cardiac output helping with enhanced blood flow, nutrient distribution and waste transportation; some of these may be beneficial in recovery from exercise. There may also be an added psychological benefit, whereby there is a reduction of the feeling of fatigue during immersion.[8–11]

The two athletes in this report presented with delayed onset muscle soreness and both had utilized the water immersion therapy. There have been several mechanisms put forth to explain the muscle soreness: accumulation of lactic acid, connective tissue damage, muscle inflammation and damage and prolonged muscle spasm. Perhaps it is a combination of these mechanisms that takes place to explain the soreness. The latter can affect performance and also increase the risk of further injury, especially if the athlete returns to the sport prematurely.[10,11]

Therefore, did the two athletes in the report do the right thing to enhance recovery?

During exercise, fluid from the blood is forced into the working muscles due to an increase in the mean arterial pressure, which thus increases the muscle volume and decreases the blood plasma fraction. Active recovery reduces this exercise-induced edema and, with an associated increase in blood flow throughout the body, may also increase the metabolism of substrates produced during exercise. There have been observations that this increased substrate metabolism reduces post exercise blood lactate following active recovery. Water immersion appears to affect a similar physiological response to active recovery without the need to expend extra energy. When a body or a large portion of it is immersed, hydrostatic pressure acts on the body’s fluid within the immersed region. Fluid from the extravascular space now moves into the vascular compartment, reducing exercise-induced increase in muscle volume and also reducing soft tissue inflammation. The blood volume increase is redistributed to increase the cardiac preload, stroke volume, cardiac output and blood flow through the body. The cardiac output increases in relation to the depth of immersion and has been observed to increase by as much as 102% during head-out immersions. These cardiovascular responses occur without any increase in energy expenditure.[2,3,9]

However, not all the effects of immersion have been shown to be positive or beneficial.

Yamane *et al*. studied matched athlete groups (immersion in cold water versus being kept at room temperature) after training and concluded that increased artery diameter and hyperthermia were transient and essential for training effects (myofiber regeneration, muscle hypertrophy and improved blood flow) to be observed. Cooling attenuates these temperature-dependent processes and appears to be disadvantageous for training.[12]

Sellwood *et al*. randomized volunteers to three, 1-min immersions in either cold water at 5°C or tepid water at 24°C and found that the ice water protocol was ineffective in minimizing markers of delayed onset muscle soreness.[4]

In a study where well-trained cyclists were compared (ice water immersion and the other group acted as control), Schiziepp *et al*. found that the brief period of cold water immersion manifests significant physiological effects that can impair subsequent cycling performance (maximum power decline 13.7% versus 4.7%, maximum heart rate decreased by 8.1% versus 2.4% compared with the respective control groups).[13]

In a trial by McDonald *et al*., where subjects were immersed to neck levels in water at 19°C after 30 min of exercise on a treadmill at 80% of maximum heart rate, there was a significant decrease in the isometric hand grip strength after 30 min of immersion compared to the group that did not have any exercise before immersion.[14]

Lactate production is evident when training or competing in sports. However, the amount produced is dependent on the duration and intensity of the exercise and the length of recovery interval. The ability to clear lactate in the recovery phase relies on the working muscle to quickly remove, tolerate and buffer H+. Hot–cold immersions may have some merit in aiding recovery if waste products are cleared faster. However, the mechanism by which active recovery promotes lactate removal is still not clearly understood. The process is complex and dependent on a range of factors such as local blood flow, chemical buffering, movement of lactate from muscle to blood as well as lactate conversion to pyruvate in the liver, muscle and heart.[15]

The use of contrast temperature water therapy by Hamlin[16] and Morton[17] showed a substantial reduction in blood lactate concentration and heart rate during the therapy. They concluded that contrast water immersion therapy is a valid method of hastening the decrease in lactate levels during recovery.

The common practice ratio of warm to cold bath duration is normally 3:1 or 4:1, with hot baths ranging from 37 to 43°C, alternating with cold baths at 12–15°C. The duration is usually 20–30 min, repeated twice daily. It has also been documented that the treatment should finish on the cold treatment to encourage vasoconstriction in the athlete.[18] There have been minimal studies comparing the effects of immersion versus just having a hot–cold shower, but it has been found that cold exposure of an approximately 1-min duration was not sufficient to significantly decrease muscle temperature following warm water immersions thus nullifying the required physiological effects.[5,9,19–24]

The two athletes described here presented with delayed onset muscle soreness post exercise. Both used only cold water immersion therapy. It is difficult to ascertain whether the therapy had a significant effect on the muscle symptoms in this case report.

## CONCLUSIONS

Training and competition creates an overload to stress the body, which in turn produces fatigue followed later by improved performance. What athletes do after their exercise and work-out regime can affect their muscle recovery. The post exercise routine can impact both fitness and sports performance. It is thus important to have an after-exercise recovery plan. Some recommendations include:

- Sufficient rest to allow for natural recovery to occur
- Gentle stretching that helps the muscle to recover faster
- A necessary cool-down period versus stopping immediately and abruptly
- A Proper balanced diet
- Adequate fluid replacement
- Proper massage

This list is sometimes followed by alternate hot and cold baths or shower and contrast water therapy. As there is still a lack of evidence with these therapies, further research will be required to investigate the different hot to cold time ratios, the appropriate mode of contrast treatment and the duration and the optimum water temperature needs to be examined to closely verify its effectiveness as a recovery modality. A holistic approach to recovery will give a better response rather than an isolated recovery technique.
